# Successful Target Temperature Management After Cardiac Arrest: A Case Report and a Review of the Literature

**DOI:** 10.7759/cureus.67986

**Published:** 2024-08-28

**Authors:** Raluca Badila, Simina Mustatea, Sandra Neamtu, Corina Roman

**Affiliations:** 1 Anesthesia and Critical Care, County Clinical Emergency Hospital of Sibiu, Sibiu, ROU; 2 Pulmonology, Clinical Pneumology Hostpital of Sibiu, Sibiu, ROU; 3 Neurology, County Clinical Emergency Hospital of Sibiu, Sibiu, ROU

**Keywords:** neuron-specific enolase, hypoxic encephalopathy, biomarkers, therapeutic hypothermia, cardiac arrest, target temperature management

## Abstract

A 20-year-old female was admitted to the hospital after a successful resuscitation from a cardiac arrest due to ventricular fibrillation. She had no prior medical history. The patient was rescued from her house, brought to the hospital with sinus rhythm, sedated, in a coma. Electrocardiography showed no modifications other than the ventricular extrasystoles. Computed tomography showed an epicranian hematoma from the fall that occurred during the cardiac arrest, a heart with a thickened interventricular septum, and the other organs being within physiological limits. Magnetic resonance imaging showed late hypoxic leukoencephalopathy with a level of the white matter of the semioval centers, radiating corona, splenium corpus callosum, and internal capsular posterior arm with extension to the cerebral peduncles. The patient achieved a good neurological outcome with target temperature management and had small neurological improvements every day after resuming spontaneous breathing. After a long intensive care and hospitalization period of six weeks, she was discharged, able to resume her societal status and be a fully recovered individual.

## Introduction

With the advancement of cardiopulmonary resuscitation (CPR) techniques and the spread of knowledge in the large population, we now have an improved success rate of CPR [1]. However, the prognosis for cardiac arrest patient survival is extremely poor, with only 10% of them surviving hospitalization. Approximately two-thirds of cardiac arrest episodes occur outside a hospital [2].

Therapeutic hypothermia, also known as targeted temperature management, involves lowering the body temperature of a patient to a mildly hypothermic state (typically 32-34°C) for a specified period, typically 12-24 hours [3-5]. This approach is based on the premise that reducing the metabolic rate and oxygen demand of the brain can mitigate the damaging effects of ischemia and reperfusion injury that occur during and after cardiac arrest [6].

Unfortunately, neuron-specific enolase (NSE) may be falsely elevated in hemolytic samples or the presence of tumors producing the protein. S100B is a routinely available glial marker often used in patients with traumatic injuries, but it is not ideal as a predictor of neurological outcomes after out-of-hospital cardiac arrest (OOHCA) [7]. According to European Society of Intensive Care Medicine guidelines, if two or more of the following are present: high, serial NSE values; no pupillary or no corneal reflexes; status myoclonus; diffuse, extensive anoxic injury on brain CT/MRI, it is certain to be a prognostic for a poor neurological outcome. After more than five days, if this is the case, it is recommended in the same guideline to start talking with the family about the course of action and explaining to them the real chance of the patient being a healthy individual.

The European Society of Intensive Care Medicine guidelines regarding temperature control post-cardiac arrest in adults recommend fever prevention for at least 72 hours in patients who are still in a coma after cardiac arrest. The guidelines also state that there is insufficient evidence to recommend or oppose temperature control at 32-36°C or early cooling after cardiac arrest. We report a case of hypertrophic cardiomyopathy leading to cardiac arrest in a family history of sudden deaths at the age of 17-20 years old, not being further investigated.

## Case presentation

After being found unconscious in her home, the patient, a 20-year-old woman, was found in cardiac arrest due to ventricular fibrillation. She was transported and admitted to the hospital. The ambulance had no information regarding how long the woman stayed in cardiac arrest without proper chest compressions being administered to her. The CPR duration was 10 minutes. The family reported that the patient was a healthy young woman, had received no treatment at home, and had no history of substance abuse. However, they did notice a family history of sudden deaths among the young people in the family but never went further with the investigations. In the first examination, the following were found: blood pressure of 90/62 mmHg; heart rate of 155 b/minute with isolated ventricular extrasystoles (VES); intubated with a SpO2 of 98%; body temperature of 36.5°C; the patient was on continuous sedation with midazolam and fentanyl, but the Glasgow coma score (GCS) before that was 4 (E1V1M2). Electrocardiography showed no modifications other than the VES. Computed tomography (CT) showed an epicranian hematoma from the fall that occurred during the cardiac arrest, and a heart with a thickened interventricular septum. Cardiac arrest was due to hypertrophic cardiomyopathy; the other organs were within physiological limits.

**Figure 1:**
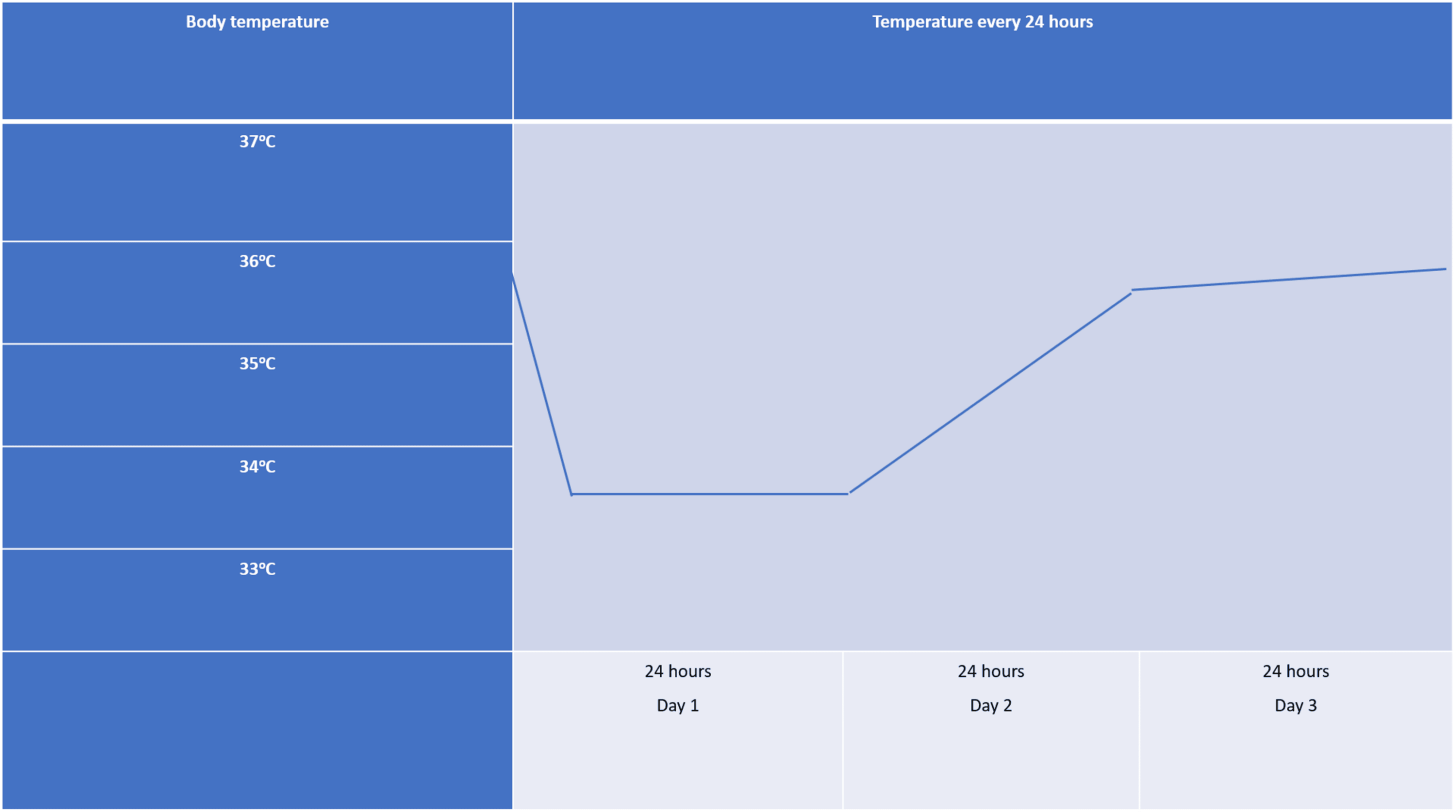
1 Body temperature from admission until day 3

The patient was treated with amiodarone for tachycardia, third-degree cephalosporin for prevention of pneumopathy associated with mechanical ventilation, and targeted temperature management. The targeted temperature management was made non-invasive by placing cooling pads on the patient, cooling down to 34°C during the first 24h, and then rewarming to 36°C for another 24h as in the protocol (Figure 1).

Among blood tests obtained on the first, third, and fifth days, NSE was collected on the first day with a value of 56 ng/mL, and again on the fifth day with a value of 18 ng/mL, which showed the same improvement in the neurological condition of the patient as seen with the clinical evaluation.

The patient was extubated on day 5, being able to breathe on her own, with a facial mask 6l/min O2. Every day, she had a clinical examination from her neurologist, cardiologist, and intensive care physician. On day 6, she had a GCS of 11 (E4V3M4), a significant improvement since day 1. She had a serum thyroid-stimulating hormone level (reference range: 0.55-4.78 mIU/L), with a surprising value of 1099 ml U/I. The patient was bradylalic at this stage. She was started on substitution therapy.

We followed a protocol for the collection of analyses, and for the imaging necessary for our case. On the first, third, and fifth days, laboratory usual analyses were sent. A CT was made on admission, repeated if necessary, and magnetic resonance imaging (MRI) was done after day 5. Also, in the protocol, it is stated that if necessary, other tests can be made at all times, for the benefit of the patient (Table 1).

**Table 1:** Laboratory analysis collection protocol CBC: Complete blood count; GCS: Glasgow coma score; NSE: Neuron-specific enolase; CT: Computed tomography; MRI: Magnetic resonance imaging; EKG: Electrocardiogram

Tests collected	Day 1	Day 2	Day 3	Day 4	Day 5
	CBC	Clinical neurologic evaluation	CBC	Clinical neurologic evaluation	CBC
	Biochemistry	GCS	Biochemistry	GCS	Biochemistry
	Coagulation	Glucose	Coagulation	Glucose	Coagulation
	CT	Temperature	CT	Temperature	MRI
	NSE	Blood pressure	Glucose	Blood pressure	NSE
	GCS		GCS		GCS
	EKG		EKG		EKG
	Clinical neurologic evaluation		Clinical neurologic evaluation		Clinical neurologic evaluation
	Blood pressure		Blood pressure		Blood pressure
	Temperature		Temperature		Temperature
	Glucose				Glucose

On the first day of admission, complete blood count (CBC) showed an average of anemia with hemoglobin of 8.9 g/dL (reference range: 12-15 g/dL) and lymphopenia of 22.1% (reference range: 25-35%). The biochemistry results revealed a high glucose level of 234 mg/dL (reference range: 70-105 mg/dL), an amylase level of 147 U/L (reference range: 28-100 U/L), and elevated TGO (AST) of 90 U/L (reference range: 11-34 U/L), TGP (ALT) of 47 U/L (reference range: 0-34 U/L), creatinine of 0.61 mg/dL (reference range: 0.5-1 mg/dL), and urea of 22 mg/dL (reference range: 15-40 mg/dL). The coagulogram showed quasi-normal values. CT showed no acute signs of intracranial pathology, right epicranial hematoma, areas of alveolar veiling apico posterior left upper lobe - contusive substrate, heart with a thickened interventricular septum, periportal edema, and in the cholecystic bed - cardiogenic substrate, segment VIII parafluid non-iodophilic liver lesion. NSE was collected on the first day with a value of 56 ng/mL. GCS before sedation on the day of admission was 4 (E1V1M2). The neurological consultation on day one was limited due to continuous sedation. Blood pressure was 90/62 mmHg. Body temperature was 36.5°C before targeted temperature management, with a target reached 24 hours later of 34°C.

On the second day, the patient was stable: maintaining a target temperature of 34°C, with the same CGS as the day before, the glucose level was 88 mg/dL.

On day three, laboratory tests revealed a low potassium of 2.86 mEq/L (reference range: 3.5-5.1 mEq/L), which was corrected by continuous potassium infusion. A C-reactive protein of 48.87 mg/L (reference range: 1-5 mg/L) was also identified, and the microbiology revealed Staphylococcus aureus in the nasal exudate. The patient started a course of third-generation cephalosporin, which was sensitive according to the antibiogram. Elevated transaminases were seen on this day. Sinus tachycardia of 135 beats per minute and no arrhythmias were found at this stage. The patient was brought back to an initial temperature of 36.5°C on this day. Still under continuous sedation with midazolam and fentanyl. Leukocytosis with neutrophilia was observed on CBC and a procalcitonin level of 0.69 ng/mL (reference range: 0-0.05 ng/mL). No bacterial growth on blood cultures and in the tracheal aspirate; Staphylococcus aureus developed, which remains sensitive to the third-generation cephalosporin. A CT was repeated at this point, with no signs of acute intracranial pathology detectable at the time of examination. According to our protocol on day four, sedation was at a lower dose so the neurologist could perform a clinical examination. Glucose levels were normal at 85 mg/dL, and the temperature was 36.7°C.

On day five, a C-reactive protein of 7.98 mg/L and a potassium of 3.6 mEq/L were the most notable items on the blood panel. Leukocytes normalized; hemoglobin was 10.7 g/dL. The sedation was interrupted on this day; the patient woke up and she was extubated with a GCS of 11 (E4V3M4). NSE on this day was at a value of 18 ng/mL.

In the following days, the patient showed significant improvement in the neurological area, reaching a GCS of 15 (E4V5M6). She was able to start enteral nutrition, and she began physiotherapy. Elevated levels of transaminases were detected on other days, which were treated with liver protectors; hemoglobin reached 11.4 g/dL.

The patient was hospitalized for six weeks and was discharged with a good neurological outcome. She is able nowadays to live a normal life without remaining immobilized after an episode of cardiac arrest with successful resuscitation. In the first 12 hours, targeted temperature management was reached, and brain protection was successfully achieved.

**Figure 2:**
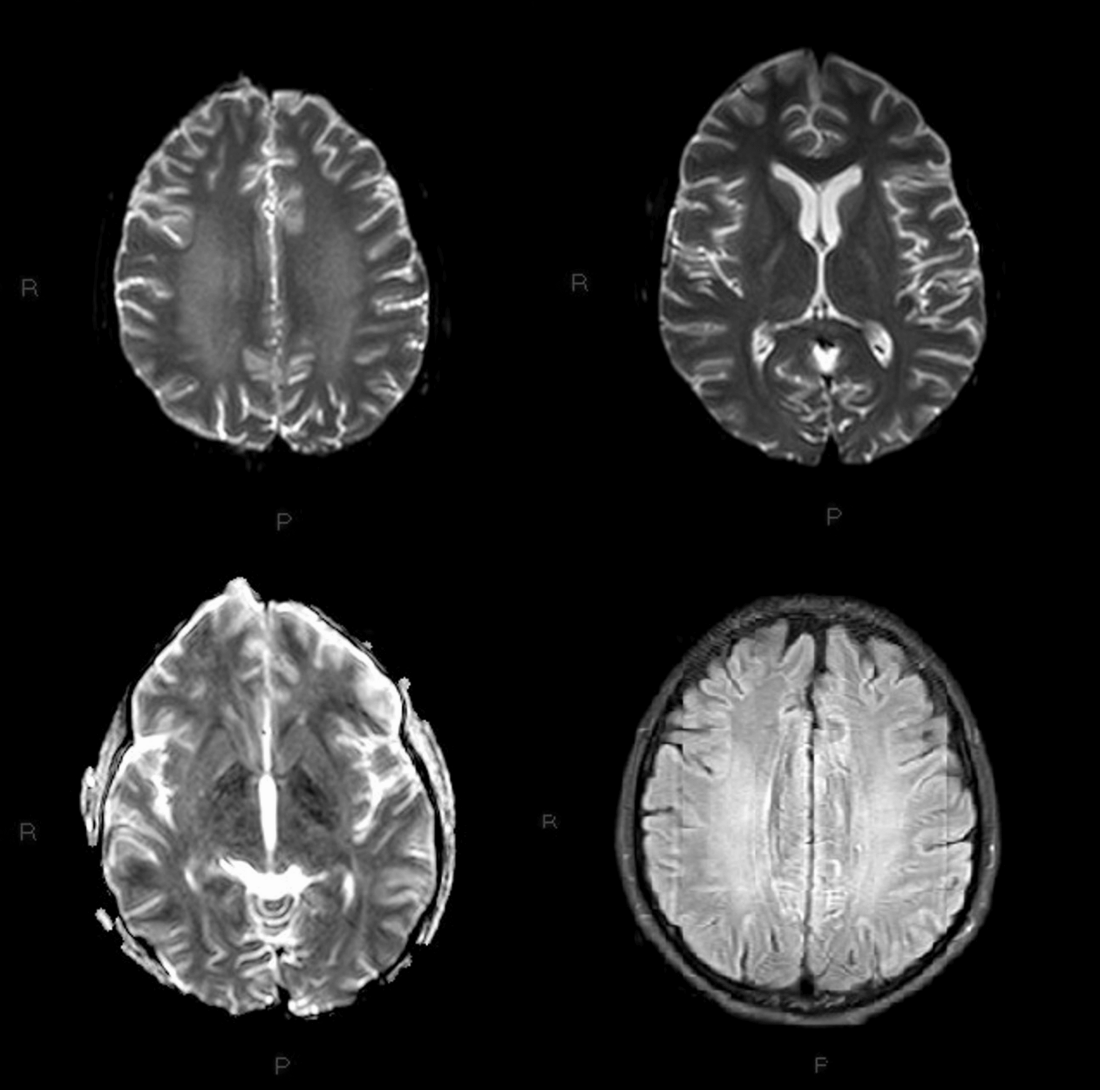
Hypoxic leukoencephalopathy with a level of white matter of the semioval centers, radiating corona, splenium corpus callosum, internal capsular posterior arm with extension to the cerebral peduncles R: Right; P: posterior

An MRI was made a week after her admission date. It showed late hypoxic leukoencephalopathy with a level of the white matter of the semioval centers, radiating corona, splenium corpus callosum, and internal capsular posterior arm with extension to the cerebral peduncles (Figure 2). The patient had small neurological improvements every day and now awaits a rehabilitation spot.

## Discussion

In this particular case, the patient was unusually young to have a cardiac arrest, making our case worth reporting. She reached target temperature management as soon as it was possible, and the neurological outcome was a very good one. The patient was prescribed beta-blockers and is currently closely monitored by her cardiologist, with regular visits at the hospital to determine if any additional treatment is needed. No arrhythmias or other complications of the heart were observed during the cooling. Shivering was observed when the patient reached 35°C but was treated easily. If arrhythmias worsen during the cooling of the patient, it is recommended by the European Resuscitation Council to keep normothermia rather than continuing with target temperature management [8].

The outcome and neurological status of OOHCA patients can improve up to six months after the event has happened [9]. A clinical examination, such as the basic neurological examination, remains the foundation of neuroprognostication. Evaluation of brainstem reflexes, the motor responses to pain, and myoclonus during the first 72 hours after cardiac arrest represent the standard evaluation [10]. However, these features can be altered by residual sedation; therefore, repeated assessments are more often necessary. The same procedure was maintained for our patient.

Bilateral absence of pupillary light reflexes being assessed at 72 hours after restoration of spontaneous circulation is a robust indicator of poor prognosis. Lack of any corneal reflexes at 72 hours is also an indicator of poor prognosis, although with lower accuracy than pupillary reflexes, especially among the patients who received sedatives or any sort of neuromuscular blockade; again, their presence is an unreliable predictor for a good outcome [11].

A reliable indicator of poor outcome after cardiac arrest is an absent or extensor response to pain at 72 hours. However, this is the physical sign most influenced by opioids, sedatives, and neuromuscular blockade; consequently, motor responses are less reliable in patients undergoing targeted temperature management [12]. Also, when assessing motor signs, it is very important to remember that while an extensor or an absent response does not specifically mean that the patient will not awaken, a localizing movement or even a flexor does not always signify that the patient will recover [13]. We could analyze the correct response of our patient after she was weaned off the ventilator and no longer needed sedation for mechanical ventilation facilitation.

A useful diagnostic tool that is also inexpensive in investigating the activity of the brain remains electroencephalogram (EEG). EEG has long been used to assess the severity of hypoxic-ischemic brain injury [14,15]. With regard to prognostication, EEG is of great help, which has made important progress in post-cardiac arrest degree of neuronal injury [16]. However, EEG interpretation can be complex and prone to subjectivity. In 2012, the American Clinical Neurophysiology Society proposed a standardized terminology for critically ill patients. The terminology was later updated in 2021 [17]. In our particular case, the patient had no EEG in her examinations because the hospital had no available machine at the moment of admission.

## Conclusions

A cardiac arrest has a significant impact on the patient's quality of life. The advantage of therapeutic hypothermia is that if the method proves to be successful, in our case it is considered a successful method, its implications will be socio-human as well as physical.

The patient will be able to resume a status within the society of which he is a part, as a recovered individual physically, and especially neurologically fit. Being able to interact and work, without being dependent on continuous care, happens in many cases where the patient responds to a cardiac arrest but remains immobilized in bed for the rest of his life due to hypoxic encephalopathy. Quality of life is a particularly important aspect of a patient's recovery. It is best to treat the illness leading to cardiac arrest, but if such a thing happens, it is always an urgent situation, and physicians need to focus on the recovery of the patient. The complexities associated with target temperature management are of great extent and can induce additional risks. It is most important to estimate those risks and evaluate if our patient can benefit from this method. 
